# Incidence and severity of self-reported chemotherapy side effects in routine care: A prospective cohort study

**DOI:** 10.1371/journal.pone.0184360

**Published:** 2017-10-10

**Authors:** Alison Pearce, Marion Haas, Rosalie Viney, Sallie-Anne Pearson, Philip Haywood, Chris Brown, Robyn Ward

**Affiliations:** 1 Centre for Health Economics Research and Evaluation, University of Technology Sydney, Sydney, New South Wales, Australia; 2 Medicines Policy Research Unit, University of New South Wales, Sydney, New South Wales, Australia; 3 NHMRC Clinical Trials Centre, University of Sydney, Sydney, New South Wales, Australia; 4 Prince of Wales Clinical School, University of New South Wales, Sydney, New South Wales, Australia; 5 University of Queensland, Brisbane, Queensland, Australia; University of Nebraska Medical Center, UNITED STATES

## Abstract

**Aim:**

Chemotherapy side effects are often reported in clinical trials; however, there is little evidence about their incidence in routine clinical care. The objective of this study was to describe the frequency and severity of patient-reported chemotherapy side effects in routine care across treatment centres in Australia.

**Methods:**

We conducted a prospective cohort study of individuals with breast, lung or colorectal cancer undergoing chemotherapy. Side effects were identified by patient self-report. The frequency, prevalence and incidence rates of side effects were calculated by cancer type and grade, and cumulative incidence curves for each side effect computed. Frequencies of side effects were compared between demographic subgroups using chi-squared statistics.

**Results:**

Side effect data were available for 449 eligible individuals, who had a median follow-up of 5.64 months. 86% of participants reported at least one side effect during the study period and 27% reported a grade IV side effect, most commonly fatigue or dyspnoea. Fatigue was the most common side effect overall (85%), followed by diarrhoea (74%) and constipation (74%). Prevalence and incidence rates were similar across side effects and cancer types. Age was the only demographic factor associated with the incidence of side effects, with older people less likely to report side effects.

**Conclusion:**

This research has produced the first Australian estimates of self-reported incidence of chemotherapy side effects in routine clinical care. Chemotherapy side effects in routine care are common, continue throughout chemotherapy and can be serious. This work confirms the importance of observational data in providing clinical practice-relevant information to decision-makers.

## Introduction

Chemotherapy is an important component of treatment for many cancers, and new anti-cancer drugs represent one of the largest areas of pharmaceutical development [1, 2]. However, the nature of chemotherapy means that while damaging cancer cells it also damages healthy cells, leading to side effects [3].

The side effects of chemotherapy affect an individual’s physical health, quality of life [4–6] and emotional state [4, 5]. The management of a side effect can include a reduction in the dose intensity of chemotherapy [7] and there is evidence that patients who receive low dose chemotherapy have reduced survival rates [8–11].

Our understanding about side effects and their frequency comes primarily from clinical trials [12]. However, this may not reflect the reality of chemotherapy side effects in routine clinical practice. For example, patients who are at risk of complications are often excluded from clinical trials, and safety monitoring may be more intensive in trials than in routine care [12]. In addition, the reporting of chemotherapy side effects in clinical trial publications is often selective, and based on the most common or most serious side effects [13, 14]. Finally, side effects in clinical trials are typically clinician reported and there is evidence that clinicians often under report the number and severity of toxicities experienced by patients [15, 16].

Observational data collected in routine clinical practice, outside clinical trials, may provide better external validity than clinical trials [17]. Previous observational studies of chemotherapy side effects have examined only a specific chemotherapy regimen [18, 19], cancer type and stage [20–23] or side effect [18, 24, 25]. There have been very few observational studies of side effects in a typical oncology practice, across a range of cancers and treatment regimens [16, 25], despite this giving data which is more representative of routine care.

The Elements of Cancer Care (EOCC) study was designed as a prospective cohort study of individuals with breast, lung or colorectal cancer undergoing chemotherapy in New South Wales, Australia [26]. The EOCC study provides the opportunity to analyse self-reported side effect information to examine the experience of chemotherapy side effects in a routine care setting. By examining the frequency of self-reported side effects in the EOCC cohort, we can obtain a more accurate estimation of the incidence of side effects in the routine care setting. As well as filling a gap in the literature, these estimates are important for clinicians and policy makers, who are usually making treatment or funding decisions about care delivered in the routine care setting and will require information about the short and long term consequences of using chemotherapy.

The objective of this study was to estimate the frequency and severity of patient-reported chemotherapy side effects in a routine-care setting across treatment centres in Australia.

## Materials and methods

### The Elements of Cancer Care study

The Elements of Cancer Care (EOCC) study design has been reported elsewhere [26], but in summary the study prospectively tracked patients undergoing chemotherapy for breast, colorectal and non-small cell lung cancer (NSCLC), and utilised information from medical and chemotherapy charts, interviews and linked administrative data. The study recruited patients from 12 cancer treatment centres in New South Wales, Australia, representing metropolitan and regional settings and the public and private hospital sectors. The eligibility criteria were that patients be aged over 18 years, able to comprehend written and spoken English (or have an interpreter available), able to give informed consent, and not participating in a clinical trial.

The primary data collection included medical-record reviews together with monthly patient interviews (programmed every 28 days) conducted by study field staff (medical students and research assistants) who were trained (by the study coordinator, using a training manual, initial shadowing and regular group review meetings) to conduct the research interviews. Data were collected on cancer treatment, health service utilisation, socioeconomic status, use of complementary therapy and a range of other factors (data collection instrument available upon request). Patient follow-up and data collection continued until either cessation of chemotherapy without recommencement within 30 days, patient withdrawal, or the census date was reached (June 11, 2010 for those recruited in 2009; May 5, 2011 for those recruited in 2010). Interviews continued for 6 months after recruitment, unless one of the above conditions were met. While extensive primary data were collected, the current report uses only the data items specifically relating to side effects. In relation to side effects, participants were asked in approximately monthly face to face or telephone interviews if they had experienced diarrhoea, vomiting, chest pain or angina, constipation, dyspnoea, fatigue, mucositis, pain or rash. These side effects were selected on the basis that they are common chemotherapy side effects which can be meaningfully reported from the patient perspective. Depression and anxiety were captured in the quality of life instrument, and are reported elsewhere. The structured questions provided the list of side effects with examples of each grade according to the NCI Common Toxicity Criteria version 4 [27] adapted to plain English (see S1 Tables for wording used). Although not a formally validated instrument, there is evidence that adapted versions of the Common Toxicity Criteria for completion by patients result in ratings consistent with those provided by their clinicians [16].

Patient written and informed consent was obtained through a face-to-face interview with study field staff. The St Vincent’s Hospital Human Research Ethics Committee approved the primary data collection. Site-specific approvals were obtained from each of the participating centres. The study is registered at Research Data Australia (Identifier 004:273).

### Analysis

Analysis was conducted for the full sample, and by cancer type. Overall frequency of side effects was calculated as the number of patients in the sample who reported the selected side effect at any grade at least once during their period of follow-up. The prevalence was calculated as the proportion of visits where each side effect was reported. The incidence rate of side effects was calculated as the number of individuals who experienced the selected side effect, divided by the total person-months of follow-up. Individuals were censored once they had experienced the selected side effect. Frequency of side effects by grade was determined using the worst grade of each side effect experienced by each individual during the follow-up period. This is consistent with the way side effects are typically reported in the clinical trial literature.

The frequency of side effects (any side effect and by each side effect) was compared between socio-demographic subgroups of gender, age (under 45 years, 46 to 65, 66), education, socioeconomic disadvantage (measured through SEIFA decile of residential postcode) [28], country of birth and cancer stage using chi-squared tests of independence, stratified for cancer type.

The cumulative incidence curve for each side effect by grade was graphed to depict patterns over time. Curves presented represent the probability of observing the side effect at that grade or higher, accounting for duration of follow-up, and was calculated using the %CUMINCID macro in SAS version 9.4.

## Results

### Demographics and clinical characteristics

There were 478 eligible individuals recruited to the EOCC study, whose full demographic, cancer and chemotherapy details have previously been published [26]. Side effect data were available from 441 individuals, with the remaining individuals having missing or incomplete data. Of the 441 individuals, there was an average 14% of missed visits (0% at the baseline visit, 17% at visit two, 10% at visit three, 16% at visit four, 14% at visit five and 13% at visit six). Median total follow-up time was 5.64 months (range 1–16.69 months), and for participants with three or more interviews (i.e. more than an initial and final interview), the average time between interviews was 36 days. Table 1 summarises the demographic and clinical characteristics of the cohort for whom side effect data were available. There were more women than men and the majority of participants were aged over 50 years. More than half the sample had breast cancer and over half had metastatic cancer.

**Table 1:** Demographic and clinical characteristics of the Elements of Cancer Care cohort.

Demographic	Breast cancer (n = 243)	Colorectal cancer (n = 142)	Lung cancer (n = 56)	Total (n = 441)
	Frequency (%)	Frequency (%)	Frequency (%)	Frequency (%)
***Gender***				
Female	240 (98.8)	56 (39.4)	30 (53.6)	326 (73.9)
Male	3 (1.2)	86 (60.6)	26 (46.4)	115 (26.1)
***Age group (years)***				
<45	46 (19.7)	7 (5.0)	2(3.6)	55 (12.8)
46–65	153 (65.4)	75 (54.0)	28 (50.0)	256 (59.7)
66+	35 (15.0)	57 (41.0)	26 (46.4)	118 (27.5)
Missing	9	3	0	12
***Higher education***				
Yes	142 (71.7)	81 (64.3)	24 (52.2)	247 (66.8)
No	56 (28.3)	45 (35.7)	22 (47.8)	123 (33.2)
Missing	45	16	10	71
***Socioeconomic disadvantage***				
High	25 (10.4)	13 (9.2)	11 (20.4)	49 (11.3)
Moderate	71 (29.6)	49 (34.8)	19 (35.2)	139 (32.0)
Low	144 (60.0)	79 (56.0)	24 (44.4)	247 (56.8)
Missing	3	1	2	6
***Country of birth***				
Australia	142 (70.3)	86 (67.7)	37 (80.4)	265 (70.7)
United Kingdom	15 (7.4)	12 (9.5)	6 (13.0)	33 (8.8)
Other	45 (22.3)	29 (22.8)	3 (6.5)	77 (20.5)
Missing	41	15	10	66
***Stage of cancer***				
Stage I	22 (9.1)	0	3 (5.4)	25 (5.7)
Stage II	80 (32.9)	6 (4.2)	2 (3.6)	88 (19.9)
Stage III	42 (17.3)	40 (28.2)	15 (26.8)	97 (22.0)
Stage IV	99 (40.7)	96 (67.6)	36 (64.3)	231 (52.4)

### Frequency, incidence and prevalence of side effects

The frequency and incidence rate of each side effect during the data-collection period is shown in Table 2. The majority of participants (86%) reported at least one side effect during the study period, and this was similar across cancers (84% breast cancer, 89% colorectal cancer, 86% of NSCL cancer). Nine percent of participants overall reported experiencing one to three side effects during the study, 10% reported experiencing four or five side effects, and 67% reported experiencing six or more side effects, and again this was similar across cancers (9%, 5% and 7% breast cancer, 9%, 18%, 61% colorectal cancer, 11%, 7%, 68% NSCL cancer).

**Table 2:** Self-reported side effects—First report of any grade of side effect during treatment.

	Breast Cancer		Colorectal cancer		NSCL Cancer		Overall		P-value†
Side effect	Frequency (%)	Incidence rate	Frequency (%)	Incidence rate	Frequency (%)	Incidence rate	Frequency (%)	Incidence rate	
Any side effect	204 (84)		126 (89)		45 (86)		378 (86)		0.43
Chest pain	26 (11)	0.03	16 (11)	0.03	9 (16)	0.05	54 (12)	0.03	0.52
Constipation	184 (76)	0.60	103 (73)	0.43	40 (71)	0.50	333 (74)	0.53	0.70
Diarrhoea	180 (74)	0.55	107 (75)	0.45	40 (71)	0.49	335 (74)	0.51	0.85
Dyspnoea	176 (72)	0.52	99 (70)	0.37	39 (70)	0.46	321 (71)	0.45	0.82
Fatigue	200 (82)	0.78	125 (88)	0.83	48 (86)	0.80	384 (85)	0.80	0.31
Mucositis	176 (72)	0.53	101 (71)	0.42	39 (70)	0.45	321 (71)	0.48	0.90
Pain	179 (74)	0.54	109 (77)	0.44	43 (77)	0.54	339 (75)	0.50	0.76
Rash	181 (74)	0.54	92 (65)	0.34	40 (71)	0.50	320 (71)	0.46	0.13
Vomiting	162 (67)	0.44	82 (58)	0.27	36 (64)	0.41	284 (63)	0.37	0.21
Anaemia†									

^†^Chi-squared test of independence for each side-effect by cancer type.

The incidence rate of any event was 0.22 events per person per month of follow-up (0.22 breast, 0.21 colorectal, 0.23 NSCL). There were no statistically significant differences in the frequency of side effects between cancer types. Each side effect, except for chest pain and vomiting, was experienced to some degree by at least 70% of participants. Fatigue was the most common (85%) side effect reported, and had the highest incidence rate with 80 individuals reporting fatigue per 100 individuals (who have not previously reported fatigue) per month of follow-up.

**Fig 1:**
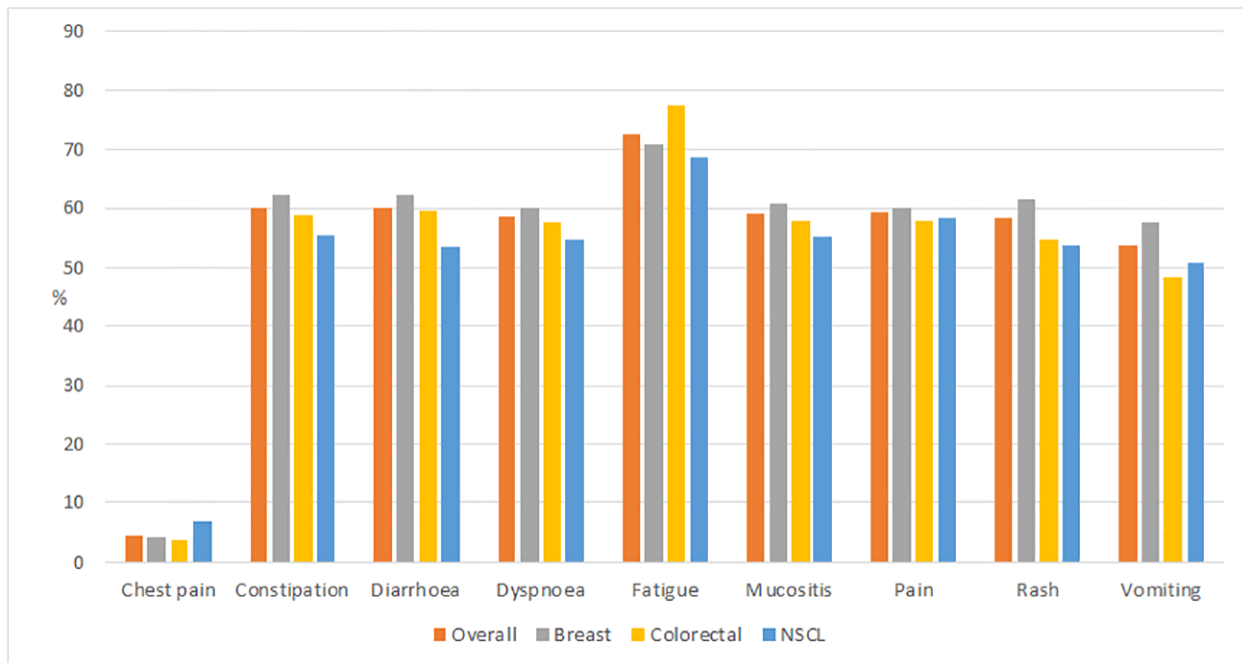
Prevalence (proportion of follow-up visits at which the specific side effect was reported) of self-reported side effects, by cancer type, during Elements of Cancer Care study period>.

The average prevalence of side effects was 54% across the cohort. Chest pain had the lowest prevalence (4% overall) while fatigue had the highest (73% overall) and prevalence was similar across the cancer types. See Fig 1 for details.

Chi-square tests of any side effect during the study period with the demographic variables (controlling for cancer site) showed no statistically significant association by gender (p = 0.10), education (p = 0.58), socioeconomic disadvantage (p = 0.47) or cancer stage (p = 0.38). Sample sizes were too small to test for the association of education and country of birth to side effects. Older participants were less likely to report a side effect, both before (p = 0.012) and after (p = 0.018) controlling for cancer type.

### Severity and cumulative incidence of side effects

Table 3 shows that most side effects were Grade I or Grade II, with relatively few instances of more-serious side effects reported. The exceptions to this are dyspnoea, fatigue and pain for which Grade III or IV events were more common. For 24% of participants overall the highest grade of side effect experienced was a mild (grade I or II) side effect, for 35% of participants it was moderate (grade III), and for 27% it was severe (grade IV). As there were no significant differences in the incidence of side effects by cancer type, these results are only shown for the combined cohort.

**Table 3:** Self-reported side effects—Worst grade reported during Elements of Cancer Care study period.

t	Grade 0		Grade I		Grade II		Grade III		Grade IV	
Side effect	Freq.	%	Freq.	%	Freq.	%	Freq.	%	Freq.	%
**Chest pain**	395	88	34	8	16	4	4	1	0	0
**Constipation**	118	26	178	40	111	25	31	7	11	2
**Diarrhoea**	116	26	208	46	99	22	21	5	5	1
**Dyspnoea**	129	29	178	40	60	13	49	11	33	7
**Fatigue**	68	15	51	11	94	21	164	37	72	16
**Mucositis**	129	29	184	41	92	20	40	9	4	1
**Pain**	111	25	157	35	66	15	82	18	33	7
**Rash**	131	29	199	44	70	16	44	10	5	1
**Vomiting**	166	37	225	50	34	8	20	4	4	1

Freq, frequency

**Fig 2:**
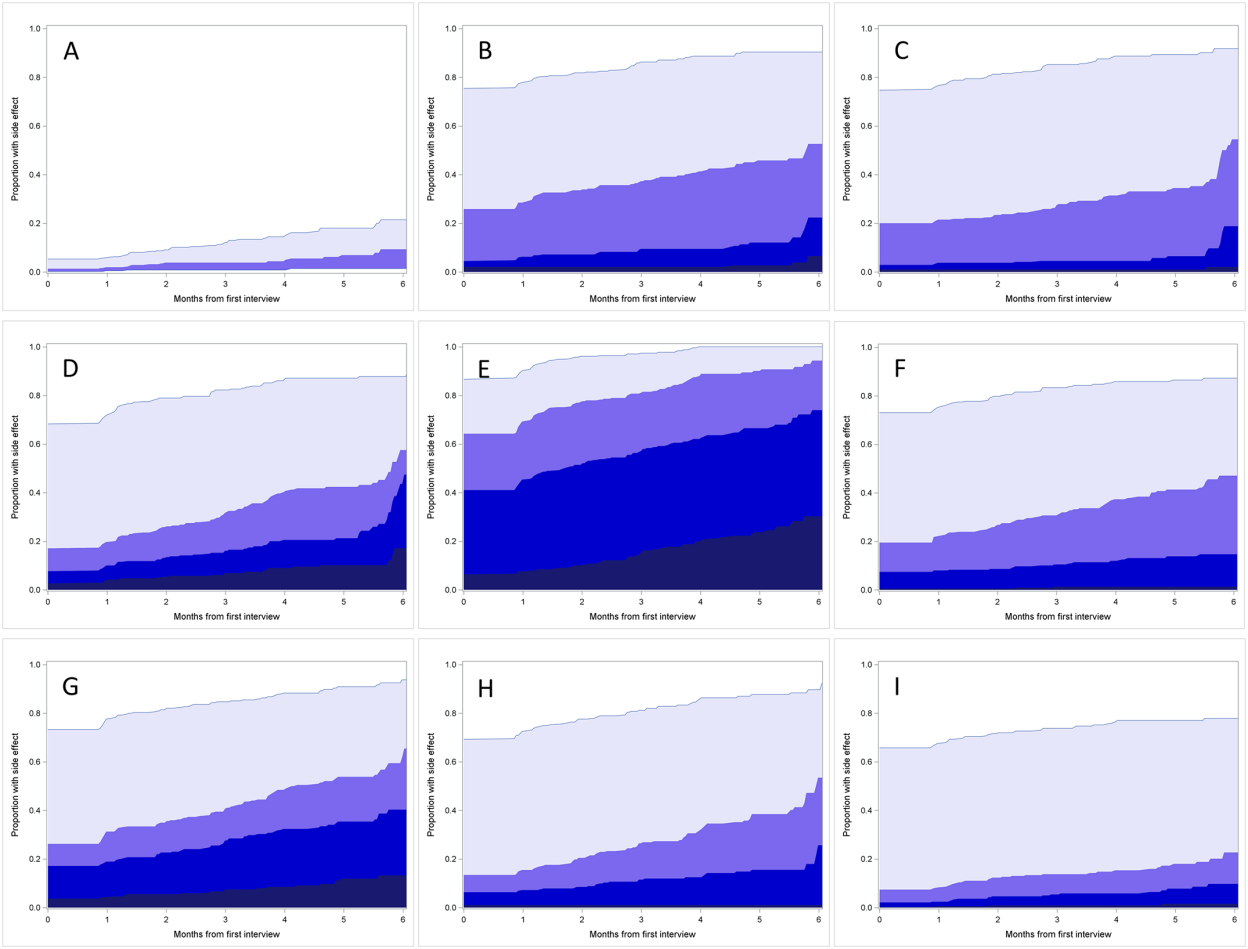
Cumulative incidence of self-reported side effects during Elements of Cancer Care study period. (A) Chest pain, (B) Constipation, (C) Diarrhoea, (D) Dyspnoea, (E) Fatigue, (F) Mucositis, (G) Pain, (H) Rash, (I) Vomiting **>**.

The cumulative incidence of each side effect is shown in Fig 2, for the 6 months after the first interview (which occurred after chemotherapy commencement). The smaller number of participants who had the full 6 months of follow-up results in the apparent jump in the graph at 6 months. As there were no significant differences in the incidence of side effects by cancer type, these results are only shown for the combined cohort. Chest pain had the lowest cumulative incidence while fatigue has the highest. While fatigue was common at each grade over time, diarrhoea and vomiting are notable for the large proportion of grade one events but relatively few more serious occurrences.

## Discussion

### Results in context

This study estimates the incidence of common chemotherapy side effects in a clinical practice setting, rather than in the context of a clinical trial. The first study of this type in Australia, we observe that over three-quarter of individuals undergoing chemotherapy in New South Wales will experience multiple side effects during their treatment, and for over 60% of people this will include a serious (grade III or IV) side effect. By presenting cumulative incidence these results give an insight into the pattern of side effects over the course of chemotherapy. The large proportion of people with mild side effects such as constipation, diarrhoea, mucositis and vomiting throughout the follow-up period is notable, as is the particularly large proportion of people reporting serious fatigue.

Although the incidence of side effects associated with chemotherapy is often reported in clinical trials of new treatments, there are few examinations of chemotherapy side effects in a community or routine care setting. A large survey of individuals undergoing chemotherapy or radiotherapy treatment for cancer conducted in the US found that 88% of the 814 respondents reported at least one side effect during their cancer treatment [25], similar to the estimate in this cohort (86%).

Consistent with other observational studies of chemotherapy side effects which have examined specific chemotherapy regimens, cancer types or side effects, our results suggest that side effects are more common in standard practice than reported in clinical trials [23, 24].

However, it can be difficult to compare incidence rates of specific side effects with these previous studies, as the duration of follow-up differs between studies. For example, a study of chemotherapy-induced nausea and vomiting in a community setting found that 36.4% of patients reported these side-effects in the five days after treatment with highly or moderately emetogenic chemotherapy [29], while our study suggests that 63% of individuals experience nausea and vomiting at some point during the median 5.64 months they were followed during treatment.

The higher rates of side effects observed over time in this study may indicate that many patients experience ongoing side effects during chemotherapy, and that for some individuals these side effects present after the first few months of treatment. For a small proportion of individuals the side effects are serious, highlighting the importance of monitoring side effects throughout treatment. The apparent jumps in cumulative incidence seen in Fig 2 are a function of the smaller numbers of participants who had the full six months of follow-up, and care should be taken with interpretation of figures in later months.

The differences in the incidence of side effects in this study compared to previous literature may also be due to the method of collecting self-reported side effects. While there is evidence that patient self-reports of side effects are consistent with clinician assessments [16], there is also evidence that different timing of and approaches to collecting side effect data can influence results [30]. Oncologists and research nurses often collect information about side effects through open ended questioning, which may result in unintended underreporting of the number and type of side effects a patient is experiencing [16, 31]. In this study, participants were provided with examples of each side effect by grade which may have encouraged them to report both a greater variety of and less severe side effects than if they had been asked open ended questions. Future research could make use of the PRO-CTCAE, the recently developed patient version of the CTCAE which now provides a validated instrument for collection of patient reported treatment toxicities [32].

While it is difficult to compare directly, the apparently higher rates of side effects seen in clinical practice compared to clinical trial reports may be explained by the strict inclusion and exclusion criteria applied in trials. Trial participants are generally younger and fitter than a typical patient seen in clinical practice [12], and it is often suggested that they may be better able to cope physically with chemotherapy and therefore less likely to experience side effects. In addition, the typical clinical trial is conducted in a large high-quality teaching hospital, where best-practice management of side effects and trial specific monitoring and follow-up are likely to reduce both the incidence and severity of any side effects [12]. However, this was not reflected in the results of this study, which suggest that younger patients were more likely to experience side effects. This may indicate that in clinical practice older patients are more likely to receive lower doses of chemotherapy, thus minimising side effects [21], although possibly also reducing treatment efficacy [8–11].

### How can these results be used?

These results highlight that side effects are common during chemotherapy, and that patient reported outcomes may identify symptoms which might not otherwise be identified. Identifying frequent side effects in patients undergoing chemotherapy in clinical practice highlights a challenge for clinicians, economic modellers and health policy makers. Oncologists and research nurses need to integrate patient-reported outcomes for symptomatic adverse events into clinical care. In addition, our results show that monitoring for side effects should continue for the duration of treatment and follow-up.

As financial pressure on the health care system mounts there is increasing demand for interventions to demonstrate cost effectiveness. Many chemotherapy cost effectiveness models are built on the basis of clinical trial data. If trial data underestimates side effects, this could have important impacts on models of cost effectiveness, as side effects may reduce patient compliance and treatment effectiveness, and increase health care costs meaning cost effectiveness would be overestimated. Future models could use our estimates of side effect frequency and prevalence as part of sensitivity analyses to test the impact of assumptions around side effect rates taken from the clinical trial literature.

In addition, policy makers are usually making decisions for the general population. While this study is too small to inform national policies directly, it points to the value of observational data because of its improved accuracy compared to administrative data [33], and greater external validity compared to clinical trials [17]. Collecting observational data is time and resource intensive [34]. While it is not feasible for an observational study to be conducted for every economic evaluation, the conduct of large, well-designed, prospective observational studies with the needs of modellers and decision makers in mind could provide valuable input to both economic models and health policy decisions.

### Strengths and limitations

This was a relatively large, prospectively designed, observational study of a cohort of individuals with cancer in New South Wales, Australia. We examined the experience of side effects across multiple cancers, rather than looking at specific chemotherapy regimens as done in the existing literature. However, our sample is limited by the small proportion of individuals with non-small cell lung cancer, who may have had different experiences of side effects to those with breast and colorectal cancer. We are also unable to differentiate those participants who are enrolled in a palliative care program. Similarly, those with other cancer types may also have different chemotherapy experiences which are not captured in this study. Without a non-cancer control group (i.e. a control group drawn from the general population without cancer and not receiving chemotherapy) or a control group with cancer but without chemotherapy, we are not able to determine what proportion of these side effects may be unrelated to chemotherapy. For example, some symptoms of the cancer itself may have been mistaken for side effects of chemotherapy. Fatigue [35] and constipation [36] are also both more common among older people, and may therefore be unrelated to treatment.

An additional limitation is that the response of the treating clinicians to the reported side effects is unknown. Some of the reported side effects may have been treated, while others may have gone unnoticed. These variations could have resulted in differences in the ongoing experience of the side effect which are not captured in the current study.

It is also possible that the retrospective self-reporting of side effects at monthly intervals may have introduced recall bias into participant responses. In the future larger, national, prospective, observational studies of individuals with a broader range of cancers and testing alternative mechanisms of side effect reporting would be valuable.

## Conclusion

This paper adds to the literature an estimate of the incidence of side effects experienced by patients undergoing chemotherapy in a routine care setting, rather than in the context of a clinical trial. It has the additional advantage over previous studies of looking across a range of cancers and treatment regimens. In this cohort, the majority of participants experienced multiple side effects and for 60% of participants at least one side effect was serious. Many patients experienced mild side effects continuously throughout the period of treatment captured in the study.

This information is useful for both clinicians and policy makers, who typically make treatment and funding decisions for standard practice, but often on the basis of potentially unrealistic clinical trials. This work also confirms the need for side effects to be collected using patient-reported methods, to be monitored throughout chemotherapy treatment, and highlights the importance of observational data in providing information for decision-makers that is relevant to the clinical practice setting.

## Supporting information

S1 File Wording of CTCAE side effects used in the Elements of Cancer Care study for patient self-report of side effects. (DOCX)
