# Supplementary material for: Incidence and severity of self-reported chemotherapy side effects in routine care: A prospective cohort study
Source: PLoS One. 2017 Oct 10;12(10):e0184360. doi: 10.1371/journal.pone.0184360 (PMC5634543; doi:10.1371/journal.pone.0184360)
Supplement: S1 File — (DOCX) [file pone.0184360.s001.docx]

**Incidence and severity of self-reported chemotherapy side effects in routine care: A prospective cohort study**

Supporting information 1: Wording of CTCAE side effects and levels when used in the Elements of Cancer Care study for patient self-report of side effects

## Dyspnoea

| **Dyspnoea** | **Recorded as…** |
| --- | --- |
| Shortness of breath at rest | Grade 4 |
| Shortness of breath on exertion, with minimal impact on activities of daily living | Grade 3 |
| No shortness of breath except on exertion, unable to walk a flight of stairs or one city block without stopping | Grade 2 |
| No shortness of breath except on exertion, able to walk a flight of stairs without stopping | Grade 1 |
| No shortness of breath | Grade 0 |

## Diarrhoea

| **Diarrhoea** | **Recorded as…** |
| --- | --- |
| Diarrhoea resulting in severe fluid losses (shock) or other severe complications | Grade 4 |
| Diarrhoea to the point where hospitalisation was required | Grade 3 |
| Mild to moderate diarrhoea, requiring IV fluids | Grade 2 |
| Mild diarrhoea | Grade 1 |
| No diarrhoea | Grade 0 |

## Constipation

| **Constipation** | **Recorded as…** |
| --- | --- |
| Constipation resulting in obstruction or other severe complication | Grade 4 |
| Constipation which significantly interfered with your usual activities | Grade 3 |
| Mild to moderate constipation occasionally interfering with your usual activities, persistent symptoms requiring the use of laxatives on most days | Grade 2 |
| Mild to moderate constipation not interfering with your usual activities, occasional symptoms with occasional use of laxatives | Grade 1 |
| No constipation | Grade 0 |

## Mucositis

| **Mucositis** | **Recorded as…** |
| --- | --- |
| Hospitalisation resulting from severe bleeding or other complication | Grade 4 |
| Extremely troublesome mouth or throat ulcers, with difficulty eating and drinking, and requiring intravenous fluids | Grade 3 |
| Mildly troublesome moth or throat ulcers, making eating or drinking difficult | Grade 2 |
| Inflamed mouth or throat, not interfering with eating | Grade 1 |
| No mouth or throat ulcers | Grade 0 |

## Vomiting

| **Vomiting** | **Recorded as…** |
| --- | --- |
| Vomiting severe enough to result in perforation or other severe complication | Grade 4 |
| Six or more episodes of vomiting in 24 hours IV fluids required | Grade 3 |
| Two to five episodes of vomiting in 24 hours may need IV fluids | Grade 2 |
| One episode of vomiting in 24 hours | Grade 1 |
| No vomiting | Grade 0 |

## Rash

| **Rash** | **Recorded as…** |
| --- | --- |
| Severe life threatening rash requiring hospital admission | Grade 4 |
| Severe rash covering more than 50% of the body | Grade 3 |
| Minimal to moderate rash, may involve blistering, covering less than 50% of the body | Grade 2 |
| Mild rash (redness of skin) anywhere on the body | Grade 1 |
| No rash | Grade 0 |

## Pain

| **Pain** | **Recorded as…** |
| --- | --- |
| Disabling pain | Grade 4 |
| Severe pain where either the pain or the medication you’re taking for the pain interferes with your daily activities | Grade 3 |
| Moderate pain where either pain or the medication you’re taking for the pain interferes with function but you can still get on with daily activities | Grade 2 |
| Minimal pain, not interfering with daily activities | Grade 1 |
| No pain in the last month | Grade 0 |

## Fatigue

| **Fatigue** | **Recorded as…** |
| --- | --- |
| Disabling fatigue | Grade 4 |
| Severe fatigue interfering with daily activities | Grade 3 |
| Minimal to moderate fatigue with some impact on activities of daily living | Grade 2 |
| Mild fatigue | Grade 1 |
| No fatigue over the month | Grade 0 |

## Chest Pain

| **Chest Pain** | **Recorded as…** |
| --- | --- |
| Chest pain or angina which resulted in a hospital admission | Admission |
| Chest pain or angina which resulted in a review in the emergency department | ED review |
| Chest pain or angina and was seen by local doctor | GP review |
| Chest pain or angina and did not seek medical advice or used own medication | No treatment |
| No chest pain or angina | No chest pain |
